# Fire safety training for workers: an investigation into how learning modalities in VR relate to performance and self-evaluations

**DOI:** 10.3389/fpsyg.2026.1740985

**Published:** 2026-02-10

**Authors:** Veronica Muffato, Marta Mazzella di Bosco, Sara Zuzzi, Daniela Pellegrini, Chiara Meneghetti

**Affiliations:** 1 Department of General Psychology, University of Padova, Padova, Italy; 2 Piazza Copernico srl, Roma, Italy

**Keywords:** immersiveness, procedural learning, safety training, stress, virtual reality

## Abstract

Learning to handle emergency situations is a fundamental goal of safety training. Virtual Reality (VR) is increasingly used to support procedural learning, yet evidence on learning conditions and learners’ perceptions is still limited in workplace fire safety training. In Study 1a, we investigated whether various active VR learning formats are more effective than traditional methods (passive video with verbal explanations) for teaching fire safety procedures (Aim 1) and examined how these methods relate to participants’ self-assessment of the experience (Aim 2). A total of 111 participants (78 females, aged 20–28) were assigned to three learning groups: (a) video-slide learning, (b) basic VR with avatar instructions, and (c) dual-mode (VR with avatar instructions plus panel information). All participants were then tested in a VR fire scenario and self-assessed their cognitive load, immersiveness, presence, motivation, and perceived stress. The results showed that both VR groups outperformed the video-slide learning group in execution time and total score, while only the basic VR group showed fewer errors than the video-slide learning group. Participants reported low cognitive load and high immersiveness, presence, and motivation with the VR experience. However, high levels of perceived stress during the simulation were associated with lower performance, confirming the negative effects of stress on learning. In Study 1b, we aimed to compare workers (*N* = 16, 9 females, 25–60 years old) with students to verify the results in a realistic context, using only the dual-mode VR learning condition. The results show no significant differences between the nonworker sample and the worker sample, suggesting that the selected procedure is applicable in a professional context. Overall, these results suggest that well-designed active VR training can enhance procedural safety learning and that psychological dimensions should be considered in its design and implementation.

## 1 Introduction

Learning and memorizing procedural knowledge during training is a crucial challenge, especially in high-risk sectors where it is necessary to prepare people to act effectively in critical situations. Traditionally, this type of learning involves the integration of theoretical lectures using teaching materials such as manuals or videos as well as practical exercises (Feng et al., 2018). Cost, safety, and resource constraints often prevent realistic scenarios from being reproduced in training, reducing exercises to low-fidelity simulations or simple demonstrations and limiting the effectiveness of learning (De Lorenzis et al., 2023; Morélot et al., 2021). Among the many fields of application that face this type of difficulty is safety, which is intended to develop knowledge required to recognize and manage potentially hazardous situations (Sacks et al., 2013), such as fires. Technologies such as Virtual Reality (VR) could be an effective solution for training (Coban et al., 2022), offering advantages such as safe training environments and solutions that enable realistic navigation and real-time interaction in complex virtual environments (Diersch and Wolbers, 2019; Feng et al., 2018; Lovreglio and Kinateder, 2020), favouring procedural knowledge (Makransky et al., 2019). In general, VR has been shown to be effective for learning and memory, as presented in the following paragraph.

### 1.1 VR features and learning

VR learning environments offer multisensory, interactive, and realistic settings that activate cognitive and behavioural processes by simulating real-life experiences (Diersch and Wolbers, 2019; Jongbloed et al., 2024). Meta-analyses show clear benefits of immersive VR over traditional and nonimmersive learning methods, with positive behavioural, cognitive, and emotional outcome. This occurs across various contexts such as education (Conrad et al., 2024; Santilli et al., 2025), vocational training (Chiang et al., 2022), medicine (Zhao et al., 2020), and industry (Radhakrishnan et al., 2021). VR appears particularly effective in strengthening procedural knowledge, including practical skills, spatial reasoning, and problem-solving (Conrad et al., 2024). Learners make fewer errors, make decisions more quickly, and execute tasks more accurately (Wismer et al., 2022), supported by real-time feedback and opportunities for iterative practice (Makransky and Petersen, 2021). The core advantage of VR over traditional, usually passive, methods is real-time interactivity (Zhao et al., 2021). Degree of interaction with the environment (e.g., sensorimotor contingencies, real-time feedback) and experiencing consequences increase cognitive strategies and make complex information more memorable (Makransky et al., 2019), thereby supporting skill transfer to real settings (Diersch and Wolbers, 2019). By contrast, outcomes for declarative knowledge appear less consistent, with studies suggesting that VR does not support factual learning as effectively (Morélot et al., 2021; Paes et al., 2021). Alongside these findings, VR relates to learners’ subjective experiences. Realistic interaction and presence can improve task focus and engagement (Buttussi and Chittaro, 2021; Makransky et al., 2019), and many learners report greater motivation, satisfaction, and self-efficacy than with traditional methods (Andersen and Makransky, 2021; Paes et al., 2021). For this immersive experience, graphic quality and interaction fidelity (the exactness with which real-world interactions can be reproduced) are important (McMahan et al., 2012). At the same time, demanding scenarios may increase stress, thereby possibly enhancing focus if moderate but impair memory and performance if excessive (Mehta et al., 2025). These features make learning in VR particularly suitable for safety training, in which acquiring and retaining procedural skills under realistic and high-pressure conditions is essential.

### 1.2 Safety trainings

Safety trainings are designed to prepare individuals to respond effectively to hazardous events, such as fires, accidents, or emergencies in confined spaces. A recent systematic review and meta-analysis (Scorgie et al., 2024) showed that VR has been increasingly applied in this field, with most studies conducted in construction safety (e.g., Afzal and Shafiq, 2021; Bhagwat et al., 2021) and fire safety (e.g., Morélot et al., 2021; Rahouti et al., 2021) and fewer applications addressing other situations, such as aviation, mining, and laboratory safety. The results indicated that VR applied to safety training has larger advantages than static materials (e.g., manuals or lectures) and smaller but still significant gains than video or desktop-based training. Most VR fire safety training studies focus on industrial or training centre environments (e.g., warehouse, hospital units, laboratory benches, vehicle engines) rather than office settings, with only a few explicitly simulating office buildings for evacuation drills (e.g., Oliva et al., 2019). In addition, the majority of studies have been conducted with students or young adults (e.g., Morélot et al., 2021; Paszkiewicz et al., 2023; Satapanasatien et al., 2021), and fewer have involved working adults or safety-critical personnel although there is evidence that VR training can also be effective in these groups (e.g., Rahouti et al., 2021, with hospital staff; Saghafian et al., 2020, with industry workers). As a result, common fire scenarios in office workplaces remain underinvestigated.

In addition, researchers have investigated subjective aspects of the training experience, such as ease of use and cognitive load (e.g., Lovreglio et al., 2021; Rahmalan et al., 2020), immersion (e.g., Morélot et al., 2021), sense of presence (e.g., Çakiroğlu and Gökoğlu, 2019), or motivation (e.g., Lovreglio et al., 2021; Rahouti et al., 2021). However, they typically examine only a single dimension at a time, sometimes using single-item questions, rather than employing a comprehensive evaluation of the psychological experience. Moreover, no study has been conducted to assess stress related to the experience even though safety training involves potentially stressful situations.

Beyond these gaps, several design features have been identified as potentially enhancing VR safety training’s effectiveness. For example, realistic environments, first-person perspectives, immediate visual or auditory feedback, and the inclusion of avatars have been proposed to consolidate procedural learning and facilitate hazard recognition (e.g., Buttussi and Chittaro, 2021; Dhalmahapatra et al., 2021; Feng et al., 2018; Jeelani et al., 2020; Pedram et al., 2020; Saghafian et al., 2020), yet these elements remain less consistently examined in VR fire safety training studies.

Taken together, these findings point to the need for further research exploring how learning modalities can be combined in VR to maximize effectiveness. Starting from Paivio’s dual coding theory (Clark and Paivio, 1991), learning can be enhanced when information is presented through verbal and nonverbal channels, for memory is supported by distinct yet interconnected pathways. Therefore, combining these modes in VR fire safety training may improve the learning experience. In particular, whether adding verbal explanations of procedures in the VR environment can further enhance learning outcomes remains an open question. Furthermore, it is important to assess the subjective evaluation of VR experience (i.e., cognitive load, immersiveness, sense of presence, and motivation) and the perceived stress experienced during the simulation as well as their potential relationship with learning procedural safety tasks.

This work consists of two consecutive studies. Study 1a was conducted on a sample of university students and involved a comparison between three learning conditions (video-slide learning, basic VR learning, and dual-mode VR learning), with the aim of examining passive versus active (using VR) procedural learning. The study also assessed the associated psychological experience.

Based on this evidence, Study 1b was conducted by comparing the student sample in the dual-mode VR learning with a sample of workers, with the aim of verifying the results’ comparability in a more practical context.

## 2 Study 1a

### 2.1 Rationale and aims

The main aim of Study 1a (aim 1) was to investigate whether learning fire safety procedures in an active and immersive manner (within a simulated VR environment using a head-mounted display) is more effective than passive learning via video-slide with verbal explanations of procedures. Two VR-based learning methods are compared to video-slide learning. We used a basic VR learning condition, in which an avatar provides instructions on the procedures to be carried out, and a dual-mode VR learning condition, in which, in addition to the avatar’s instructions, verbal information was displayed on panels. Aim 2 was to compare the latter two VR learning conditions. Because VR-based training relies on the processing of three-dimensional spatial information (Diersch and Wolbers, 2019), a Mental Rotation Test (MRT) was administered to ensure group comparability in visuospatial ability. Additionally (Aim 3), we investigated the subjective experience of learning in VR by considering factors such as cognitive load, immersiveness, sense of presence, and motivation. We also examined the role of stress experienced during the simulation. Studies have shown that these variables can relate to training outcomes. Presence and motivation can enhance engagement and retention whereas excessive cognitive load or stress may hinder performance (e.g., Chittaro and Buttussi, 2015; Makransky and Petersen, 2021). For instance, in medical and military VR training contexts, higher perceived stress has been associated with increased workload and reduced training benefits (Lackey et al., 2016; Mühling et al., 2023), although there is less evidence for these effects in fire-safety contexts specifically.

We expected that

*(H1)* Participants who receive training using VR (basic and dual-mode) will perform significantly better on safety-related procedural tasks than with video-slide learning (e.g., Scorgie et al., 2024).

*(H2)* Participants in the dual-mode VR learning (VR learning plus verbal informative panels) may achieve higher performance scores than participants in a basic VR learning condition without verbal panes, based on the dual coding theory, which suggests that learning is improved when visual and verbal information is provided (Clark and Paivio, 1991).

*(H3)* The relationship between subjective factors (cognitive load, immersiveness, sense of presence, and motivation) and perceived stress with VR performance will be examined, with the possibility that these factors may be related to actual performance (Buttussi and Chittaro, 2021; Wismer et al., 2022). In particular, we hypothesized that lower perceived stress would be associated with greater procedural learning (e.g., Chittaro and Buttussi, 2015; Mühling et al., 2023).

### 2.2 Methods

#### 2.2.1 Participants

A total of 111 university students (78 females), aged between 20 and 28 years, took part in the study in exchange for course credits. Participants were assigned to one of three groups (*N* = 39 in the basic VR learning, *N* = 36 in the dual-mode VR learning, *N* = 36 in the video-slide learning) per balancing criteria based on gender, visuospatial abilities (score obtained in a mental rotation task), and previous experience in safety training courses. This allocation process resulted in three groups that were homogeneous in composition. The inclusion criterion for the participants was not suffering from motion sickness, and the exclusion criterion was a history of psychiatric, neurological, or other illnesses causing cognitive, visual, auditory, or motor deficits diagnosed by a professional. For the correlation analysis, the sample size, determined using the “pwr” library in R, indicated that 84 participants were sufficient to achieve a power of 0.80, an anticipated medium correlation (*r* = 0.30), and a significance level of *p* < 0.05. For the multivariate Bayesian regression model, the sample size approach was also balanced with practical, ethical, and feasibility considerations. This ensured a sufficient number of observations to capture the complexity of the relationships between variables and to guarantee that the collected data were informative enough to allow for a robust updating of priors.

The Ethical Committee for Psychological studies of University of Padova (No. 1091-a) approved this study. Participants were informed of the study aims and gave their written informed consent in accordance with the Declaration of Helsinki (World Medical Association, 2013).

#### 2.2.2 Materials

##### 2.2.2.1 Personal and informational questions (created *ad hoc*)

It includes items on age, gender, manual dominance, previous courses attended on safety, susceptibility to motion sickness, and familiarity with devices (one question: “Indicate your familiarity with controllers and joysticks”).

##### 2.2.2.2 Visuospatial task: mental rotations test

It consists of 10 items in which a target figure is presented, flanked by four alternatives from which to identify those two representing the same figure, rotated in space (De Beni et al., 2014). One point is awarded for each item if both correct answers are identified, and their sum is calculated (maximum score = 10). The test has good reliability (current sample Cronbach’s alpha = 0.82).

##### 2.2.2.3 Factual knowledge about safety procedures test (created ad hoc)

It consists of 14 questions (see Supplementary materials) assessing knowledge of safety procedures, each with four answer choices (one correct), selected from a pool of questions actually used in industry safety training (e.g., “In the event of a continuous alarm signal, what should you do after leaving your workplace and following the instructions of the personnel in charge?”). Two parallel forms of similar difficulty have been implemented. The score is the sum of the correct answers (maximum score = 14).

##### 2.2.2.4 Fundamentals of safety procedure slides (created ad hoc)

To ensure that participants started with the same understanding of basic knowledge on safety procedures, informative slides have been created specifically on the main procedures to follow during an electrical fire, how to prevent it, and on fire extinguishers (reading time: 5 min).

##### 2.2.2.5 Safety training (learning phase)

**Figure 1:**
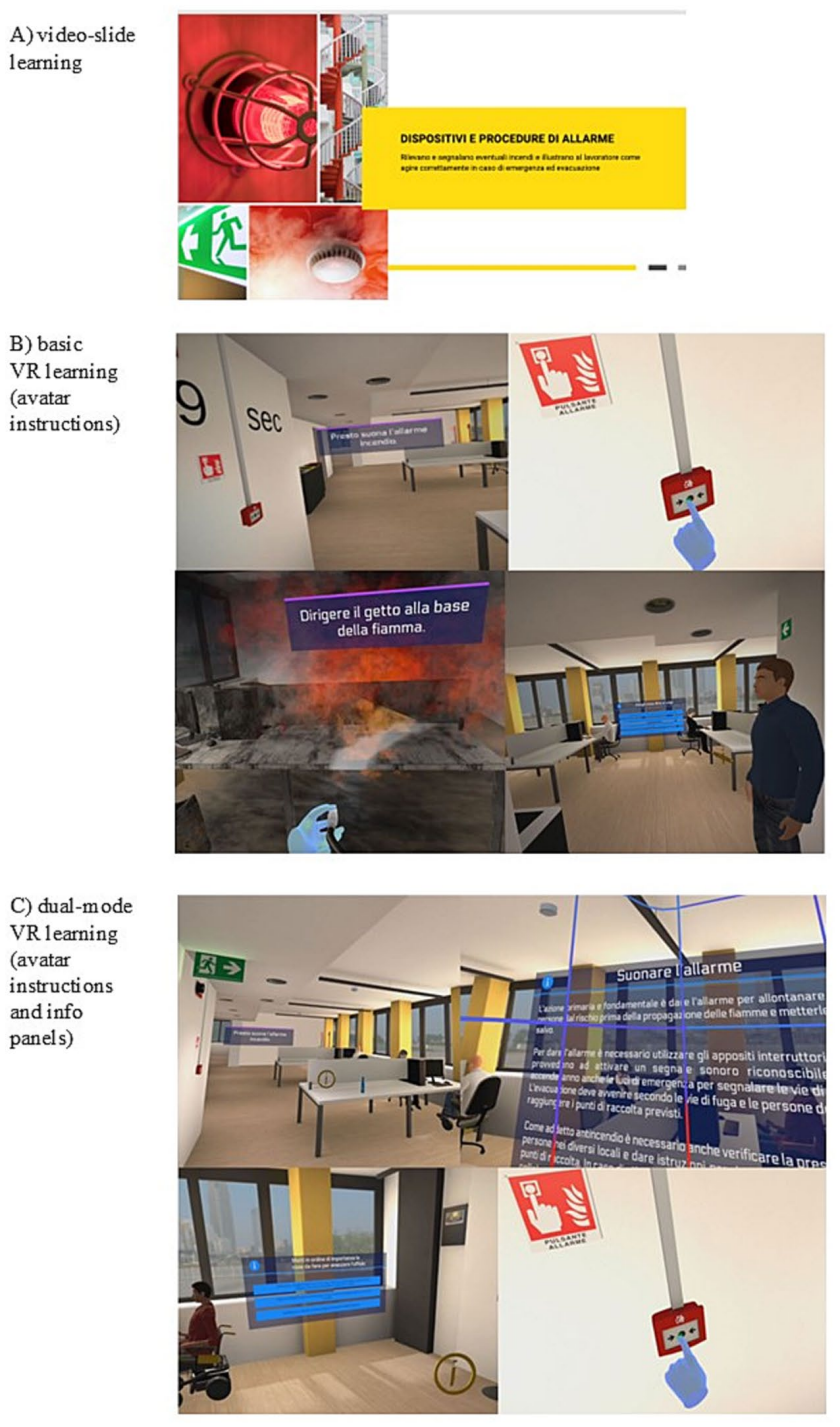
Examples of safety training in video-slide learning group **(A)**, basic VR group **(B)**, and dual-mode group **(C)**.

*Safety training from video-slide learning.* Learning took place through the use of multimedia content presented in a two-dimensional format (PC desktop), consisting of slides with a narration voice covering the following topics: electricity in the workplace, alarm devices and procedures, workplace safety regulations, fire extinguishers, and firefighting procedures (duration: 20 min). See Figure 1.

*VR-learning conditions.*

*VR environment setup and how it works*. An immersive virtual environment was designed (using the WEAVR Creator based on Unity 3D), modelling realistic three-dimensional environments, defining interactive logic, integrating immediate feedback, and configuring the dynamic behaviour of avatars. The simulation was compatible with VR headset mode, and all user actions—errors, times, and interactions—were tracked according to the xAPI standard and sent to a learning record store for analysis and evaluation of the experience to simulate a fire situation inside an office and displayed using a headset (Meta Quest 3) and hand controllers for navigation and interaction with elements in the environment. The learning phase consists of managing a minor electrical fire, during which participants are required to correctly complete a sequence of actions: recognizing the degree of fire risk and alerting colleagues, locating the source of the fire, disconnecting the electrical panel, choosing the correct fire extinguisher, removing the safety pin, holding the hose and pressing the delivery valve, directing the spray at the base of the flame, opening the windows, and drawing up a near-miss report.

*Basic VR learning.* In this group, participants followed verbal instructions provided by an avatar (see Figure 1). Instructions were presented only once; however, if a participant made an error, the avatar would repeat the instruction until the task was performed correctly.

*Dual-mode VR learning.* In this group, participants also followed verbal instructions provided by an avatar but had additional access to interactive pop-up panels and aids integrated into the virtual environment (see Figure 1). As in the basic VR condition, verbal instructions were presented only once but were repeated if an error occurred during task execution. The interactive panels appeared near the specific task areas and could be opened to provide additional procedural information and theoretical explanations to assist the user in completing the step requested by the avatar.

##### 2.2.2.6 Assessment of learning (testing phase)

For all groups, procedure assessment took place in VR using a Meta Quest 3 headset and hand controllers for navigation and interaction. The task involved managing a medium-sized electrical fire and rescuing an injured person by completing a sequence of actions: identify the danger, sound the alarm, alert and evacuate colleagues, prepare and wet protective equipment, select the correct extinguisher, call to the injured person, open the door, extract the injured person, remove the extinguisher safety pin, hold the hose and press the valve, aim at the base of the flame, exit through the emergency door, and reach the assembly point. The basic VR learning and video-slide learning groups carry out the simulation in the basic version, in which interaction is guided exclusively by instructions provided by an avatar. In the dual-mode VR learning group, participants have access also to verbal information panels and aids integrated into the virtual environment. Maintaining the same interaction features during testing phase was intended to assess performance as it would occur in real training contexts.

Performance was assessed based on the number of errors and the time required to complete the procedure. Additionally, a total score for the learning procedure was calculated from execution time and the number and severity of errors to replicate how performance is typically assessed in applied safety training scenarios in companies (see Supplementary materials for details).

##### 2.2.2.7 Evaluation of the VR experience questionnaire (created *ad hoc*)

It consists of 25 items aimed at assessing cognitive load (e.g., “The virtual reality experience greatly improved my understanding of the procedures covered”, 6 items; adapted from *Multidimensional Cognitive Load Scale for Virtual Environments,* Andersen and Makransky, 2021), immersiveness (e.g., “I was so immersed in this virtual reality experience that I lost track of time”, 3 items; adapted from *User engagement scale*, O’Brien et al., 2018), sense of presence (e.g., “The movements within the virtual reality experience were natural”, 6 items; adapted from *Presence questionnaire*, Witmer and Singer, 1998), and motivation (e.g., “It was important for me to perform well during the virtual reality experience”, 10 items; adapted from *Intrinsic Motivation Inventory,* Ryan, 1982) during the VR experience. See all the items in the Supplementary materials. Responses are given on a 6-point Likert scale, from 1 = *not at all agree* to 6 = *strongly agree*. The score is the sum of the item responses (maximum score = 150). The total score on the questionnaire has good reliability (current sample Cronbach’s alpha = 0.88).

##### 2.2.2.8 Perceived stress during the simulation scale (created ad hoc)

It consists of a 6-item scale measuring the level of perceived stress and emotions experienced during the simulation (e.g., “I felt unable to control the situation during the simulation”; adapted from *Perceived Stress Scale −10*, Cohen et al., 1983). See all items in the Supplementary materials. The answers are given on a 5-point Likert scale, from 1 = *not at all* to 5 = *very much*. The score is the sum of the item responses (max. 30). The total of the questionnaire has good reliability (current sample Cronbach’s alpha = 0.86).

#### 2.2.3 Procedure

Participants completed two sessions: one online (10 min) and one in the lab (60 min). In the online session, they filled out the personal information questionnaire and the Mental Rotations Test. Gender, Mental Rotations Test scores, and prior safety course attendance were used to balance assignment to the three groups. In the lab session, participants first completed a factual knowledge test on safety procedures, then viewed slides on basic safety fundamentals before beginning the learning phase.

In the basic and dual-mode VR groups, learning took place in VR using a Meta Quest 3 headset and controllers. Participants received device instructions before starting. The simulation involved managing a minor electrical fire (to limit stress during learning) by completing a prescribed sequence of actions; errors required repeating the step until correct, and the simulation ended only after all avatar-guided steps were completed (about 20 min). The video-slide group learned via multimedia content on a PC desktop (20 min). After a 5-min break, the assessment phase began. All groups are asked to extinguish a medium-sized electrical fire and rescue an injured person (to assess performance under higher pressure) by following a sequence of actions (about 20 min) in VR simulation. The video-slide group received device instructions before starting the assessment. At the end, all participants retook the factual knowledge test (parallel form), then completed questionnaires on the self-evaluation of VR experience and perceived stress during the simulation. Additional measures were collected but are not reported here.

#### 2.2.4 Data analysis

Analyses were run in R (R Core Team, 2025). First, descriptive statistics and correlations between variables were calculated. Correlations were calculated with the correlation() package (Makowski et al., 2020). Given the nonnormal distribution of the variables, for continuous variables, we used Spearman correlations; for dichotomous categorical variables, we used the Phi coefficient (*φ*) based on Pearson’s chi-square; for categorical variables that were not both dichotomous, we used Cramér’s V based on Pearson’s chi-square. For combinations of categorical and continuous variables, the Kruskal–Wallis test was used, supplemented by Cliff’s delta as a measure of effect size to quantify the strength of the association through stochastic dominance.

We employed a correlation matrix analysis to explore the bivariate relationships among all variables of interest. This helped us identify variables significantly correlated with our dependent variables: errors, time, and total score. This initial analysis, combined with our focused interest in the research problem, guided the selection of individual predictive variables for inclusion in the subsequent model.

Subsequently, we evaluated the effects of group and perceived stress on the measured performances (errors, time, and total score), using a multivariate Bayesian regression model from the brms package (Bürkner, 2017). The errors were modelled with a Poisson distribution (log link, see Supplementary Figure S1), time with a Gamma distribution (log link, see Supplementary Figure S2), and the normalized total scores with a beta regression (logit link; see Supplementary Figure S3), each predicted by group and the standardized perceived stress scores (see correlation table below).

### 2.3 Results

#### 2.3.1 Descriptives and correlations between variables

See descriptives divided by group in Table 1 and correlations in Table 2. Evaluation of the VR experience showed high mean values across all groups suggesting a generally positive evaluation. We verified that 97% of participants in the dual-mode VR group opened more than half of the information panels.

**Table 1:** Means and standard deviations of the variables of interest divided by group (Study 1a).

Variable	Video-slide learning		Basic VR learning		Dual-mode VR learning	
	*M*	DS	*M*	DS	*M*	DS
Age	21.58	1.56	21.66	1.29	21.36	0.99
Mental rotation test (max. 10)	5.08	3.01	4.89	3. 21	5.29	2.83
Device familiarity (max. 9)	5.14	2.55	4.50	2.56	5.86	2.25
Factual knowledge pre (max. 14)	11.61	1.23	11.46	1.06	11.44	1.31
Factual knowledge post (max. 14)	12.36	1.13	11.47	1.53	12.20	0.89
Evaluation of the VR experience scale (max. 150)	102.89	14.49	103.45	22.40	106.84	15.63
Perceived stress scale (max. 30)	13.89	5.59	11.92	4.58	13.50	5.13
Learning phase in VR: Errors	/	/	6.78	1.69	6.94	2.38
Learning phase in VR: Time	/	/	14.15	8.29	13.90	6.68
Learning phase in VR: Total score	/	/	94.31	12.77	97.61	16.48
Assessment phase: Errors	9.94	2.72	7.13	2.56	8.94	2.66
Assessment phase: Time	22.10	10.75	13.10	4.30	14.87	5.46
Assessment phase: Total score	988.05	47.23	1026.58	28.48	1037.53	42.68

**Table 2:** Correlations between variables (Study 1a).

Variable	1	2	3	4	5	6	7	8	9	10	11	12	13	14
1. Gender (male)	–													
2. Gender (female)	–	–												
3. Age	0.27	−0.27	–											
4. Mental Rotation Test	**0.37** ^**^	**−0.37** ^***^	0.03	–										
5. Device Familiarity	**0.59** ^***^	**−0.59** ^***^	0.07	0.32	–									
6. Factual knowledge (pre)	0.08	−0.08	−0.08	−0.05	0.09	–								
7. Factual knowledge (post)	0.04	−0.04	0.04	0.30	0.08	−0.05	–							
8. Evaluation of VR Experience	0.25	−0.25	0.15	0.16	0.29	0.02	0.22	–						
9. Perceived stress	−0.19	0.19	−0.22	0.00	−0.23	0.17	0.02	**−0.37** ^**^	–					
10. Video-slide group	0.02	−0.02	0.03	0.00	0.01	0.05	0.19	−0.04	0.11	–				
11. Basic VR group	0.01	−0.01	0.07	−0.03	−0.20	−0.04	−0.27	−0.04	−0.16	–	–			
12. Dual-mode VR group	0.02	−0.02	0.10	0.03	0.19	0.00	0.08	0.08	0.06	–	–	–		
13. Assessment phase: errors	−0.20	0.20	−0.07	−0.15	−0.04	0.06	0.25	−0.11	**0.39** ^**^	0.32	**−0.39** ^**^	0.07	–	
14. Assessment phase: time	−0.11	0.11	0.06	0.20	−0.06	−0.06	0.07	−0.10	**0.33** ^*^	**0.47** ^***^	−0.32	−0.15	**0.50** ^***^	–
15. Assessment phase: total score	0.21	−0.21	0.02	0.19	0.16	0.10	−0.12	0.21	−0.32	**−0.46** ^***^	0.15	0.31	**−0.77** ^***^	**−0.70** ^***^

Spearman *ρ* was reported for associations between continuous variables, Phi (*φ*) was used for dichotomous categorical variables, Cramér’s V was used for non-dichotomous categorical variables, and the Kruskal–Wallis test with Cliff’s delta was used for associations between continuous and categorical variables. Significant correlations in bold type. ** *p* < 0.01; *** *p* < 0.001.

The evaluation of the VR experience correlated with perceived stress (using Spearman with *ρ* = −0.37, *p* < 0.01), indicating that participants with greater evaluation of VR perceived lower stress. Perceived stress correlated with assessment phase errors (using Spearman with *ρ* = 0.39, *p* < 0.01) and time (using Spearman with *ρ* = 0.33, *p* < 0.05), indicating that people who perceived higher stress made higher errors and longer time when testing their learning. Concerning the correlation by learning group, the video-slide learning group was correlated with assessment phase total score (using Cliff’s *δ* with *ρ* = −0.46, *p* < 0.001) and time (using Cliff’s *δ* with *ρ* = 0.47, *p* < 0.001), indicating that participants in the video-slide condition achieved lower scores and required more time during the assessment phase compared to the other groups. The basic VR learning group was negatively correlated with assessment phase errors (using Cliff’s *δ* with *ρ* = −0.39, *p* < 0.01), indicating that participants in this condition committed fewer errors during the assessment phase.

#### 2.3.2 Multivariate Bayesian model

**Figure 2:**
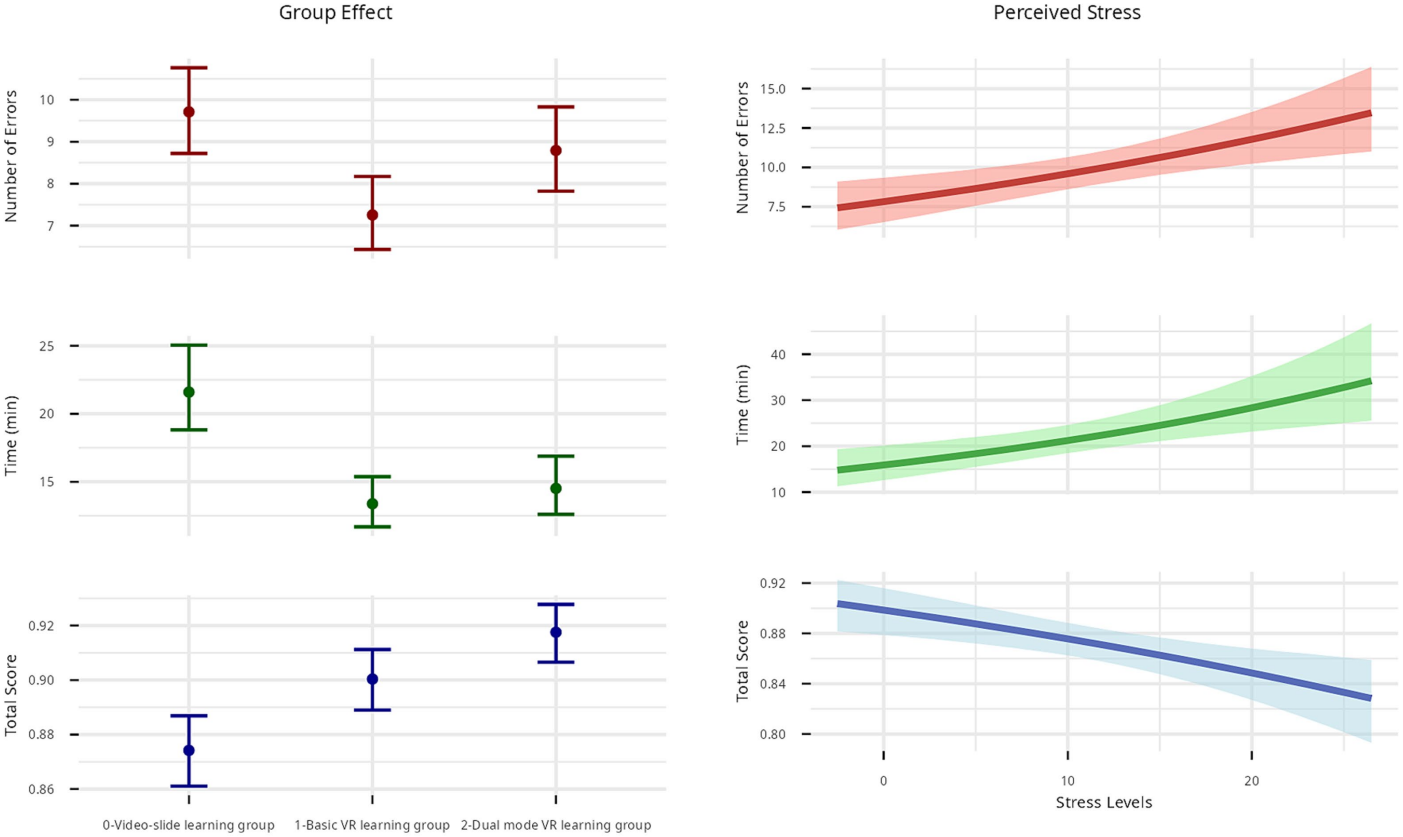
Multivariate Bayesian model effects (Study 1a).

Concerning H1 and H2, the basic VR group made 25.2% fewer errors than the video-slide learning group (95% CI [−36.2, −12.2%]). The dual-mode group showed no significant differences in errors compared to the video-slide learning group. Run times were significantly shorter in the basic group (−38.1%) and the dual-mode group (−33.0%) than in the video-slide learning group (basic: 95% CI [−49.3, −24.4%], dual-mode learning: 95% CI [−45.1, −17.3%]). The total score was significantly higher for the basic group (Δ = +0.26; 95% CI [0.09, 0.43]) and the dual-mode group (Δ = +0.47; 95% CI [0.29, 0.65]) than for the video-slide learning group. Concerning H3, Higher levels of stress (inserted as predictors, given it is the only subjective variable related to the performance) are associated with a significant increase in errors (+2.02% per unit of stress), an increase in execution time (+3.04%), and a decrease in score (Δlog-odds = −0.02 Δlog-odds = −0.02; 95% CI [−0.04, −0.01]). See effects in Table 3 and Figure 2.

**Table 3:** Bayesian model parameters estimates (Study 1a).

Outcome	Predictor	Estimate	95% CI
Errors	Basic VR vs. Video-slide (% change)	−25.2	[−36.2, −12.2%]
	Dual-mode vs. Video-slide (% change)	−9.5%	[−22.1, 5.1%]
	Stress (% per unit)	+2.02	[1.00, 3.04%]
Time	Basic VR vs. Video-slide (% change)	−38.1	[−49.3, −24.4%]
	Dual-mode vs. Video-slide (% change)	−33.0	[−45.1, −17.3%]
	Stress (% per unit)	+3.04	[1.00, 5.13%]
Total score	Basic VR vs. Video-slide (Δ log-odds)	+0.26	[0.09, 0.43]
	Dual-mode vs. Video-slide (Δ log-odds)	+0.47	[0.29, 0.65]
	Stress (Δ log-odds)	−0.02	[−0.04, −0.01]

Estimates represent posterior means. Credible intervals not including zero indicate a statistically meaningful effect.

### 2.4 Discussion

We investigated the effectiveness of VR for learning safety procedures in a simulated fire emergency. The results confirmed H1: VR training significantly improved performance (execution time and total score) from training to assessment, compared to video-slide learning. However, no significant differences emerged between basic VR learning and dual-mode VR learning, and the dual-mode VR group did not significantly differ from the video-slide learning group in terms of errors (H2 not supported). All groups reported positive evaluations of cognitive load, immersiveness, sense of presence, and motivation whereas perceived stress negatively affected performance, being linked to more errors, longer execution times, and lower scores (H3 partially supported).

These findings show that active VR enhances procedural learning in fire safety training, compared to traditional passive instruction, through active interaction, immediate feedback, and realistic simulation. No relationship was found between VR training and factual knowledge, replicating previous literature (Makransky et al., 2019). Contrary to expectations based on Paivio’s dual coding theory (Clark and Paivio, 1991), the dual-mode VR condition did not yield additional benefits. Possible explanations include redundancy of information and distraction caused by the supplementary panels, a greater reliance on avatar guidance, and the structured nature of the task, which may have reduced the need for additional support. Although performance in both VR conditions was high, the dual-mode format may still be promising for more complex tasks or longer training cycles (Wismer et al., 2022). Individual differences also mattered: Participants reporting higher stress made more errors, took longer to complete the procedure, and achieved lower scores. In immersive VR safety training, stress should be considered a central design and evaluation dimension (Mehta et al., 2025). Although moderate arousal may support engagement, exceeding the optimal threshold compromises learning, consistent with evidence from emergency training contexts linking higher perceived stress to increased workload and reduced training benefits (e.g., Lackey et al., 2016; Mühling et al., 2023). This underscores the need to measure and regulate stress systematically.

To sum up, the results obtained confirmed the need to adopt multidimensional evaluation approaches in VR-based training contexts, including objective performance indicators but also subjective dimensions (Wismer et al., 2022).

Because the training is intended for workers, Study 1b included a sample of workers.

## 3 Study 1b

### 3.1 Rationale and aims

We aimed to compare the dual-mode VR learning condition, as a chosen learning modality, between students and workers. The purpose is to assess whether this procedure produces comparable outcomes in a more practical context, thereby supporting the results’ generalizability and practical relevance. We expected that the student group’s performance would be comparable to that of the worker group, indicating similar effectiveness of the dual-mode VR learning condition across both populations.

### 3.2 Methods

#### 3.2.1 Participants

A total of 16 working adults (mean age = 40.36, SD = 12.86; females = 9) were compared to the 36 students in the dual-mode VR learning case of Study 1a.

Workers were distributed across the following occupational sectors: accounting and management (*n* = 7, 43.8%), innovation and technology development (*n* = 4, 25%), education (*n* = 2, 12.5%), hospitality (*n* = 2, 12.5%), and law enforcement (*n* = 1, 6.2%). With regard to education level, most participants held a high school diploma (*n* = 3, 18.75%); others reported lower-secondary education (*n* = 3, 18.8%), a bachelor’s degree (*n* = 3, 18.8%), or a master’s/postgraduate degree (*n* = 7, 43.75%).

The exclusion criteria included susceptibility to motion sickness, and a history of psychiatric, neurological, or other illnesses causing cognitive, visual, auditory, or motor deficits diagnosed by a professional.

#### 3.2.2 Materials and procedure

The same material and procedure for dual-mode VR learning were applied as in Study 1a. The same scales on evaluation of the VR experience and perceived stress were administered.

#### 3.2.3 Data analysis

Analyses were conducted in R (R Core Team, 2025). First, descriptive statistics were calculated. Nonparametric Wilcoxon rank-sum tests were conducted to compare the student group and worker group. Correlational analyses (as in Study 1a) were conducted to examine whether similar patterns emerged in relation to psychological factors.

### 3.3 Results

See descriptives divided by group in Table 4. A Wilcoxon rank-sum test indicated no significative difference in performance during the VR experience between the worker group and the student group (*W* = 0.34). This suggests that workers obtained similar performance levels as students in the learning phase (error: *p* = 0.97, time: *p* = 0.64, total score: *p* = 0.90) and the testing phase (error: *p* = 0.44, time: *p* = 0.13, total score: *p* = 0.19) (Table 4).

**Table 4:** Means and standard deviations of the variables of interest divided by workers and students (Study 1b).

Variable	Workers		Students	
	*M*	DS	*M*	DS
Age	39.58	13.01	21.36	0.99
Mental rotation test (max. 10)	4.03	3.35	5.29	2.83
Device familiarity (max. 9)	5.06	2.93	5.86	2.25
Factual knowledge pre (max. 14)	11.97	1.30	11.44	1.31
Factual knowledge post (max. 14)	12.00	1.39	12.20	0.89
Evaluation of the VR experience scale (max. 150)	116.13	15.73	106.84	15.63
Perceived stress scale (max. 30)	10.84	5.25	13.50	5.13
Learning phase in VR: errors	7.06	1.69	6.94	2.38
Learning phase in VR: time	13.14	6.02	13.86	6.68
Learning phase in VR: total score	98.01	12.14	97.61	16.48
Assessment phase: errors	8.47	3.02	8.94	2.66
Assessment phase: time	12.23	4.98	14.87	5.46
Assessment phase: total score	1053.00	37.31	1037.53	42.68

Similar correlational patterns were observed between the student and worker samples across the variables examined (see Supplementary Table S1 for combined sample and Supplementary Table S2 for worker sample).

### 3.4 Discussion

In Study 1b, we examined whether the performance of students in the dual-mode VR training could be replicated in a worker sample. The results showed no significant differences between groups in task completion time, errors, total score, or the subjective evaluation of VR (cognitive load, immersiveness, sense of presence, motivation) and perceived stress. This provides preliminary and exploratory evidence that this fire safety training may generalize to workers. However, the small worker sample is a limitation, and the findings should be interpreted with caution. Future research should include larger and more diverse occupational samples to further validate these findings and explore potential moderators, such as prior training experience, job-specific demands, and perceived stress by gender. Nevertheless, VR safety training seems promising for successful application in real-world organizational contexts (Santilli et al., 2025).

## 4 General discussion and conclusions

In the present studies, we evaluated VR’s effectiveness for learning fire safety procedures in emergency scenarios and extended previous evidence on VR in safety training (Scorgie et al., 2024). In Study 1a, we compared three learning conditions (video-slide, basic VR, dual-mode VR) among university students and investigated the evaluation of the VR experience and perceived stress. In Study 1b, we tested whether the dual-mode VR condition generalized to a worker sample.

The results showed that active VR training produced fewer errors and shorter run times as well as higher total scores than the passive video-slide learning, confirming its advantages for procedural learning (Makransky et al., 2019). Importantly, these differences should be interpreted in light of the fact that the VR conditions involved active practice, guided rehearsal, and close similarity between training and evaluation tasks. As such, the observed performance advantages cannot be attributed to VR as a representational format alone, but rather to the instructional features embedded in the VR training. High subjective ratings of cognitive load, immersiveness, sense of presence, and motivation also supported VR’s experiential benefits (Buttussi and Chittaro, 2021). This aligns with findings that interactive VR environments strengthen procedural knowledge, spatial reasoning, and problem solving, particularly when combined with real-time feedback (Conrad et al., 2024; Makransky and Petersen, 2021).

Contrary to expectations based on dual coding theory (Clark and Paivio, 1991), the dual-mode VR learning did not outperform the basic VR version. Possible explanations include redundancy or distraction from the supplementary panels; greater reliance on avatar guidance; and the structured nature of the task, which may not have required additional support (Morélot et al., 2021). Nevertheless, the dual-mode format remains promising because optional, on-demand aids can promote autonomy, personalization, and inclusive learning, potentially supporting more complex tasks or longer training cycles (Wismer et al., 2022).

Perceived stress emerged as a critical factor: Higher stress was associated with more errors, longer execution times, and lower scores. Consistent correlational patterns across students and workers suggest that the psychological mechanisms underlying VR learning are robust. The results on perceived stress align with recent work, suggesting that demanding VR scenarios can raise stress, possibly thereby enhancing focus if moderate but hindering performance if excessive (Mehta et al., 2025). These findings also extend evidence from other emergency training contexts (e.g., medical and military; Lackey et al., 2016; Mühling et al., 2023) to fire safety training. These findings underscore the importance of designing immersive training to balance realism with emotional regulation, using adaptive features, gradual exposure, or user-paced guidance to optimize learning outcomes (Meshkat et al., 2024).

These findings show that VR fire safety training offers scalable, repeatable learning experiences without the risks and costs of real-world simulations. When well designed, VR can enhance procedural skills even under simulated pressure. This study deepens our understanding of how to learn fire safety procedures and stresses the importance of integrating psychological factors into instructional design. Adaptive features or onboarding phases can help users acclimate to immersive environments, and companies should implement VR fire safety programs with these precautions to strengthen workplace preparedness.

Although this study provides important insights, several limitations should be noted. First, a key methodological limitation is that the VR training is active and was compared with passive video-slide learning. Future studies should compare interactive VR with interactive PC-based training or passive VR with passive video learning. Second, future research should include larger and more diverse samples of professionals from various industries to improve generalizability. Third, long-term learning retention should be assessed. Fourth, assessment conditions were not fully equivalent across groups, as the dual-mode VR group retained access to instructional panels during testing, which may have influenced performance measures; future studies should assess all groups under identical conditions. Fifth, baseline procedural knowledge should be assessed. Sixth, given the emotional and cognitive factors identified here, it would be valuable to explore adaptive VR systems that respond dynamically to user stress levels, performance, or prior experience. Finally, incorporating physiological indicators, such as heart rate or skin conductance, could provide more objective insights into user engagement and stress management in immersive environments.

In conclusion, these studies show that active VR fire safety training improves procedural performance, compared with traditional passive methods, and may yield comparable outcomes for students and workers. High ratings of immersiveness, presence, motivation, and cognitive load confirm its experiential benefits. However, perceived stress emerged as a key negative factor; therefore, this aspect should always be considered and minimized where possible. When carefully designed and implemented, VR makes safety more learnable, with important implications for companies and organizational contexts.

## Data Availability

The datasets presented in this study can be found in online repositories. The names of the repository/repositories and accession number(s) can be found at: Zenodo repository: https://zenodo.org/records/16607753.
