# Supplementary material for: Fire safety training for workers: an investigation into how learning modalities in VR relate to performance and self-evaluations
Source: Front Psychol. 2026 Feb 10;17:1740985. doi: 10.3389/fpsyg.2026.1740985 (PMC12929460; doi:10.3389/fpsyg.2026.1740985)
Supplement: Supplementary file 1 [file Data_Sheet_1.pdf]

Supplementary Material

1 Supplementary Methods

1.1. Factual knowledge about safety procedures test (created ad hoc from an industry safety training)

**Form A**		
Topic	English version	Italian Version
Evacuation (E)	In the event of a continuous alarm signal, what should you do after leaving your workplace and following the instructions of the personnel in charge?<br><br>A. Use fire extinguishers to try to put out the fire.<br>B. Search other rooms for colleagues who may be in danger.<br>C. Go to the assembly point and remain there until the alarm has ceased.<br>D. Take those who are intoxicated outside and then return inside to rescue other injured persons.	In caso di segnale di allarme continuo, che cosa è necessario fare, dopo aver abbandonato il posto di lavoro e aver seguito le indicazioni degli addetti?<br><br>A. Utilizzare gli estintori per provare a spegnere l'incendio.<br>B. Cercare nelle altre stanze gli altri colleghi in pericolo.<br>C. Raggiungere il punto di raccolta e rimanervi sino al cessato allarme.<br>D. Portare gli intossicati all'esterno e poi tornare all'interno per recuperare altri feriti.
Procedure (P)	In the event of a fire, what should the workers in charge do?<br><br>A. Wait for the fire brigade to arrive without intervening.<br>B. Only extinguish the fire if it is large, ignoring smaller fires.<br>C. Manage the emergency by following the evacuation plan and using fire-fighting equipment.<br>D. Direct colleagues towards the lift for a quicker evacuation.	In caso di incendio, che cosa devono fare i lavoratori incaricati?<br><br>A. Attendere l'arrivo dei Vigili del Fuoco senza intervenire.<br>B. Spegnere l'incendio solo se di grandi dimensioni, ignorando i casi più piccoli.<br>C. Gestire l'emergenza seguendo il piano di evacuazione e utilizzando i dispositivi antincendio.<br>D. Dirigere i colleghi verso l'ascensore per un'evacuazione più rapida.
Fire (F)	What do you need to know before using a fire extinguisher?<br><br>A. The stability of its pressurised tank.	Che cosa occorre conoscere prima di utilizzare un estintore?<br><br>A. La stabilità del suo serbatoio sotto pressione.

	B. The classes of fire for which it is suitable.<br>C. Its operating temperature.<br>D. Its extinguishing capacity.	B. Le classi di fuoco per cui è adatto.<br>C. La sua temperatura d’uso.<br>D. La sua capacità estinguente.
Fire (F)	During ignition, what should workers designated to implement firefighting measures do?<br><br>A. They should attempt to extinguish the fire at any cost.<br>B. They must attempt to extinguish the fire by assisting the fire brigade.<br>C. They must attempt to extinguish the fire by moving as far away from the scene of the fire as possible.<br>D. They must attempt to extinguish the fire without endangering their own safety or that of others.	In fase di ignizione, i lavoratori designati per l’attuazione della lotta antincendio che cosa devono fare?<br><br>A. Devono tentare di spegnere il principio d’incendio a qualsiasi costo.<br>B. Devono tentare di spegnere il principio d’incendio aiutando i vigili del fuoco.<br>C. Devono tentare di spegnere il principio d’incendio allontanandosi il più possibile dal luogo dell’incendio.<br>D. Devono tentare di spegnere il principio d’incendio senza pregiudizio per la propria o altrui incolumità.
Evacuation (E)	What information must be clearly communicated to the fire brigade?<br>A. The exact address of the location where the incident occurred.<br>B. The names of those present at the scene of the fire.<br>C. The contact details of the company's CEO.<br>D. The presence of carcinogenic substances at the scene of the fire.	Che cosa è necessario comunicare in modo chiaro ai Vigili del fuoco?<br>A. L’indirizzo preciso del luogo dove è accaduto l’evento.<br>B. Il nome dei presenti nel luogo dell’incendio.<br>C. Un recapito dell’a.d. dell’azienda.<br>D. La presenza di sostanze cancerogene nel luogo dell’incendio.
Fire (F)	How should a fire extinguisher be used correctly?<br><br>A. Direct the jet towards the base of the flames with horizontal movements.<br>B. Direct the jet towards the top of the flames with vertical movements.<br>C. Spray the contents directly onto the flames without moving.<br>D. Only act once the fire has spread over a large area.	Come si utilizza correttamente un estintore?<br><br>A. Dirigere il getto verso la base delle fiamme con movimenti orizzontali.<br>B. Dirigere il getto verso la parte alta delle fiamme con movimenti verticali.<br>C. Spruzzare il contenuto direttamente sopra le fiamme senza movimenti.<br>D. Agire solo dopo che l'incendio ha coinvolto una vasta area.
Fire (F)	When is it advisable to immediately abandon an attempt to extinguish a	Quando è consigliabile abbandonare immediatamente un tentativo di

	fire?<br><br>A. When the fire extinguisher runs out.<br>B. When the flames become too high and uncontrollable.<br>C. When there are no other people present.<br>D. When the smoke temporarily subsides.	spegnimento?<br><br>A. Quando si finisce la carica dell'estintore.<br>B. Quando le fiamme diventano troppo alte e incontrollabili.<br>C. Quando non ci sono altre persone presenti.<br>D. Quando il fumo diminuisce temporaneamente
Evacuation (E)	When evacuating a building in case of fire, what is the correct behaviour?<br><br>A. Use the lift to get down faster.<br>B. Follow the marked escape routes without running or causing panic.<br>C. Wait until the fire is completely extinguished before evacuating.<br>D. Take personal belongings before leaving the building.	Quando si deve evacuare un edificio in caso di incendio, qual è il comportamento corretto?<br><br>A. Utilizzare l'ascensore per scendere più velocemente.<br>B. Seguire le vie di fuga segnalate senza correre o creare panico.<br>C. Attendere che l'incendio sia completamente spento prima di evacuare.<br>D. Prendere oggetti personali prima di lasciare l'edificio.
Procedure (P)	What should the coordinator do after a fire emergency?<br><br>A. Simply report the incident verbally<br>B. Wait for the next scheduled inspection<br>C. Write a detailed report and check the condition of the fire-fighting equipment<br>D. Ignore the incident if no one was injured	Cosa deve fare il coordinatore dopo un'emergenza incendio?<br><br>A. Limitarsi a comunicare verbalmente l'accaduto<br>B. Attendere il prossimo controllo programmato<br>C. Redigere un rapporto dettagliato e verificare lo stato dei presidi antincendio<br>D. Ignorare l'accaduto se non ci sono stati feriti
Fire (F)	What is the correct sequence for using a fire extinguisher?<br><br>A. Press the lever directly<br>B. Shake the extinguisher and spray<br>C. Spray randomly in all directions<br>D. Remove the pin, hold the dispenser, press the lever	Quale è la sequenza corretta per l'utilizzo di un estintore?<br><br>A. Premere la leva direttamente<br>B. Scuotere l'estintore e spruzzare<br>C. Spruzzare casualmente in tutte le direzioni<br>D. Togliere la spina, impugnare l'erogatore, premere la leva
Evacuation (E)	During a fire evacuation, what is the	Durante l'evacuazione per incendio,

	correct behaviour?<br><br>A. Use the emergency stairs and remain calm.<br>B. Use the lift to evacuate more quickly.<br>C. Wait for help while remaining in your office.<br>D. Run quickly, pushing others aside to get out first.	quale comportamento è corretto?<br><br>A. Utilizzare le scale di emergenza mantenendo la calma<br>B. Utilizzare l'ascensore per evacuare più velocemente<br>C. Attendere i soccorsi rimanendo nel proprio ufficio<br>D. Correre velocemente spingendo gli altri per uscire prima
Practical Scenario (PS)	You see thick smoke coming out of a technical room. You have a filter mask and fireproof gloves at your disposal. How do you proceed?<br><br>Option A: You put on full PPE before approaching to assess the situation.<br>Option B: You quickly enter the room to check the situation without wasting time with PPE.	Vedi fumo denso uscire da un locale tecnico. Hai a disposizione una maschera con filtro e guanti ignifughi. Come procedi?<br><br>Opzione A: Indossi i DPI completi prima di avvicinarti per valutare la situazione<br>Opzione B: Entri rapidamente nel locale per verificare la situazione senza perdere tempo con i DPI
Practical Scenario (PS)	During an inspection, you notice multiple electrical sockets connected in series and accumulated paper materials nearby. How do you handle the situation?<br><br>Option A: Report the situation but wait for the next scheduled inspection<br>Option B: Arrange for the immediate removal of the materials and the reorganisation of the electrical connections	Durante un'ispezione noti più prese elettriche collegate a cascata e materiale cartaceo accumulato vicino. Come gestisci la situazione?<br><br>Opzione A: Segnali la situazione ma attendi il prossimo controllo programmato<br>Opzione B: Disponi l'immediata rimozione del materiale e la riorganizzazione delle connessioni elettriche
Practical Scenario (PS)	During an emergency, you notice that some evacuation signs are covered by stacked materials. How do you proceed?<br><br>Option A: Immediately remove the obstacles and report the critical issue to the person in charge.<br>Option B: Ignore the problem and focus solely on the emergency at	Durante un'emergenza noti che alcuni cartelli di evacuazione sono coperti da materiale accatastato. Come procedi?<br><br>Opzione A: Rimuovi immediatamente gli ostacoli e segnali la criticità al responsabile<br>Opzione B: Ignori il problema concentrandoti solo sull'emergenza in corso

	hand.	
**Form B**		
Topic	English version	Italian Version
Evacuation (E)	If the emergency plan calls for a two-stage evacuation, what should you do in the event of a continuous alarm signal?<br><br>A. Leave your workstation immediately.<br>B. Call the fire brigade.<br>C. Equip yourself with a fire extinguisher.<br>D. Remain at your workstation and wait for instructions.	Se il piano di emergenza prevede un’evacuazione in due fasi, in caso di segnale di allarme continuo che cosa è necessario fare?<br><br>A. Abbandonare immediatamente la postazione di lavoro.<br>B. Chiamare i Vigili del fuoco.<br>C. Munirsi di estintore.<br>D. Rimanere sul posto di lavoro in attesa di indicazioni.
Procedure (P)	When should you wear a mask or respiratory protection device during a fire?<br><br>A. Only if the fire is very close.<br>B. Only if you can see the fire.<br>C. It is not necessary to wear one during a practical drill.<br>D. Whenever there is smoke in the environment.	Quando si deve indossare una maschera o un dispositivo di protezione respiratoria durante un incendio?<br><br>A. Solo se l’incendio è molto vicino.<br>B. Solo se si vede il fuoco.<br>C. Non è necessario indossarla durante una prova pratica.<br>D. Ogni volta che c’è presenza di fumo nell’ambiente.
Fire (F)	Workers designated to implement firefighting measures must attempt to extinguish the fire…<br><br>A. ... using any means at their disposal.<br>B. ... only if they are able to do so.<br>C. ... by throwing water on the flames before using the fire extinguisher.<br>D. ... as soon as the active combustion phase begins.	I lavoratori designati per l’attuazione della lotta antincendio devono tentare di spegnere il principio d’incendio…<br><br>A. … utilizzando qualsiasi mezzo a loro disposizione.<br>B. … solo qualora siano in grado di farlo.<br>C. … gettando acqua sulle fiamme prima di usare l’estintore.<br>D. … appena inizia la fase di combustione attiva.
Fire (F)	How can you tell which classes of fire a fire extinguisher is suitable for?<br><br>A. Colour.<br>B. Shape.	Da che cosa si capisce per quali classi di fuoco è adatto un estintore?<br><br>A. Colore.<br>B. Forma.

	C. Type of diffuser or hose.<br>D. Information on the label.	C. Tipo di diffusore, o manichetta.<br>D. Indicazioni sull'etichetta.
Fire (F)	How can you recognise a safe escape route during a fire?<br><br>A. It must always be lit and free of obstacles.<br>B. It must be the shortest route, regardless of signage.<br>C. It is the one closest to the lift.<br>D. It does not matter which one it is, the important thing is to get out quickly.	Come si riconosce una via di fuga sicura durante un incendio?<br><br>A. Deve essere sempre illuminata e priva di ostacoli.<br>B. Deve essere la via più breve, indipendentemente dalla segnaletica.<br>C. È quella più vicina all'ascensore.<br>D. Non importa quale sia, l'importante è uscire velocemente.
Fire (F)	Which of the following information can be included on the fire extinguisher label?<br><br>A. Duration of the extinguishing agent contained.<br>B. Combined effect of use with other types of fire extinguishers.<br>C. Possibility of use on live electrical equipment.<br>D. Tank dimensions.	Quale di queste informazioni può essere riportata sull'etichetta dell'estintore?<br><br>A. Durata dell'agente estinguente contenuto.<br>B. Effetto combinato dell'utilizzo con altre tipologie di estintori.<br>C. Possibilità di utilizzo su impianti in tensione.<br>D. Dimensioni del serbatoio.
Fire (F)	What is the main risk posed by smoke from a fire?<br>A. It reduces visibility and can cause panic.<br>B. It can cause intoxication and loss of consciousness.<br>C. It can hinder the operation of fire extinguishers.<br>D. It can reduce the temperature of the environment.	Qual è il rischio principale del fumo prodotto da un incendio?<br>A. Riduce la visibilità e può causare panico.<br>B. Può intossicare e causare perdita di coscienza.<br>C. Può ostacolare il funzionamento degli estintori.<br>D. Può ridurre la temperatura dell'ambiente.
Evacuation (E)	Which of the following is a correct fire prevention measure?<br><br>A. Keep escape routes clear and periodically check fire-fighting equipment<br>B. Store paper materials near heat sources<br>C. Use multiple power strips to	Quali tra queste è una corretta misura di prevenzione incendi?<br><br>A. Mantenere sgombre le vie di esodo e controllare periodicamente i presidi antincendio<br>B. Accumulare materiale cartaceo vicino alle fonti di calore<br>C. Utilizzare prese multiple a cascata per

	optimise electrical connections<br>D. Keep all fire doors open to facilitate passage	ottimizzare le connessioni elettriche<br>D. Tenere aperte tutte le porte antincendio per facilitare il passaggio
Procedure (P)	During a fire emergency, what is the correct sequence of actions?<br><br>A. Raise the alarm, begin evacuation, call for help<br>B. Attempt to extinguish the fire yourself, call colleagues, evacuate<br>C. Gather personal belongings, call for help, exit<br>D. Wait for the fire to subside before taking action	Durante un'emergenza incendio, quale sequenza di azioni è corretta?<br><br>A. Dare l'allarme, iniziare l'evacuazione, chiamare i soccorsi<br>B. Tentare lo spegnimento da soli, chiamare i colleghi, evacuare<br>C. Raccogliere gli effetti personali, chiamare i soccorsi, uscire<br>D. Aspettare che l'incendio si plachi prima di agire
Fire (F)	When responding to a fire, what type of respiratory protection is necessary?<br><br>A. Mask with a specific filter for smoke<br>B. No protection if the fire is small<br>C. Wet handkerchief over the mouth<br>D. Hold your breath to avoid inhaling smoke	In caso di intervento su un principio d'incendio, quale protezione delle vie respiratorie è necessaria?<br><br>A. Maschera con filtro specifico per fumi<br>B. Nessuna protezione se l'incendio è piccolo<br>C. Fazzoletto bagnato sulla bocca<br>D. Trattenere il respiro per evitare di inalare fumi
Evacuation (E)	How can you recognise a sign indicating an emergency exit?<br><br>A. Red sign with an arrow<br>B. Yellow sign with the word “Exit”<br>C. Blue sign with a pictogram of a fire extinguisher<br>D. Green sign with a white pictogram and a directional arrow	Come si riconosce un cartello che indica una via di fuga?<br><br>A. Cartello rosso con freccia<br>B. Cartello giallo con scritta "Uscita"<br>C. Cartello blu con pittogramma di un estintore<br>D. Cartello verde con pittogramma bianco e freccia direzionale
Practical Scenario (PS)	During the evacuation, you notice that a colleague wants to go back to retrieve important personal belongings. How do you respond?<br><br>Option A: You accompany them to retrieve their belongings.<br>Option B: You stop them, explaining that the priority is to get to safety.	Durante l'evacuazione, noti che un collega vuole tornare indietro per recuperare effetti personali importanti. Come ti comporti?<br><br>Opzione A: Lo accompagni a recuperare gli effetti personali<br>Opzione B: Lo fermi spiegando che la priorità è mettersi in salvo

Practical Scenario (PS)	(SP) During a meeting in the conference room, you hear the fire alarm and notice smoke coming from the corridor. As the emergency coordinator, what do you do?<br><br>Option A: Continue the meeting while waiting for confirmation of the actual emergency<br>Option B: Stop the meeting, activate the evacuation procedure and check the source of the smoke	(SP) Durante una riunione in sala conferenze, senti l'allarme antincendio e noti del fumo provenire dal corridoio. Come coordinatore delle emergenze, cosa fai?<br><br>Opzione A: Continui la riunione aspettando conferma dell'emergenza reale<br>Opzione B: Interrompi la riunione, attivi la procedura di evacuazione e verifichi la fonte del fumo
Practical Scenario (PS)	(SP) After a fire was put out with fire extinguishers, some colleagues would like to return to the premises. How do you handle the situation?<br><br>Option A: Prevent access until the fire brigade has verified that conditions are safe.<br>Option B: Allow controlled re-entry to retrieve personal belongings.	(SP) Dopo un principio d'incendio domato con estintori, alcuni colleghi vorrebbero rientrare nei locali. Come gestisci la situazione?<br><br>Opzione A: Impedisci l'accesso fino alla verifica delle condizioni di sicurezza da parte dei VVF<br>Opzione B: Permetti il rientro controllato per recuperare oggetti personali

1.2. Total score calculation details

The total score was based on the scoring method used in industry safety trainings. Specifically, in all conditions the total score was obtained by subtracting 5 points for each minor error, 10 points for each medium error, and 15 points for each serious error from the starting score (initial score = 1065). There was also a time penalty of 2.5 points for every minute of delay beyond the time allocated for each task, up to a maximum of 50 points (for delays more than 20 minutes). Finally, only in the dual mode VR group a bonus is awarded based on the use of the additional information provided by panels, accessible within the environment: in the participant consulted at least 50% of the information panels (with a minimum stay of 15 sec each), 30 points are awarded; if they accessed all of them (100%). The points awarded were reduced to 15. No bonus was awarded if the information panels was no opened.

1.3. Evaluation of the VR experience questionnaire (created ad hoc)

Evaluation of the VR experience questionnaire

Instruction: Below you will find some statements relating to the experience that has just ended. Read each statement carefully and rate your level of agreement on a scale of 1 to 6, where 1 = strongly disagree and 6 = strongly agree.

	1 = Strongly Disagree	2	3	4	5	6 = Strongly Agree

	English Version	Italian Version
1	The virtual reality experience presented procedures that I found very complex (R)	L'esperienza in realtà virtuale ha proposto procedure che ho percepito come molto complesse (R)
2	The virtual reality experience dealt with concepts and definitions that I found very complex (R)	L'esperienza in realtà virtuale ha trattato concetti e definizioni che ho percepito come molto complessi (R)
3	The virtual reality experience greatly improved my understanding of the procedures covered	L'esperienza in realtà virtuale ha migliorato notevolmente la mia comprensione delle procedure trattate
4	The instructions used in the virtual reality experience were full of unclear content (R)	Le istruzioni utilizzate nell'esperienza in realtà virtuale erano piene di contenuti non chiari (R)
5	It was difficult to find information relevant to the activities proposed in the virtual reality experience (R)	È stato difficile trovare le informazioni rilevanti ai fini delle attività proposte nell'esperienza in realtà virtuale (R)
6	Elements in the virtual environment made learning unclear (R)	Gli elementi nell'ambiente virtuale hanno reso l'apprendimento poco chiaro (R)
7	I was so immersed in this virtual reality experience that I lost track of time	Ero così coinvolto in questa esperienza in realtà virtuale che ho perso la cognizione del tempo
8	I blocked out external distractions during the virtual reality experience	Ho bloccato le distrazioni esterne a me durante l'esperienza in realtà virtuale
9	.During the virtual reality experience, I was aware of things happening around me (R)	Durante l'esperienza in realtà virtuale ero consapevole delle cose che accadevano intorno a me (R)
10	In the virtual reality experience, my interactions with the environment felt natural	Nell'esperienza in realtà virtuale, le mie interazioni con l'ambiente mi sono sembrate naturali
11	I was engaged during the experience in the virtual reality environment	Ero coinvolto durante l'esperienza nell'ambiente in realtà virtuale
12	The movements within the virtual reality experience were natural	I movimenti all'interno dell'esperienza in realtà virtuale erano naturali

13	My experiences within the virtual environment felt consistent with my experiences in the real world	Le mie esperienze all'interno dell'ambiente virtuale mi sono sembrate coerenti con le mie esperienze nel mondo reale
14	I experienced a time lag between my actions and the response of the virtual reality experience that was consistent with my expectations	Ho sperimentato un intervallo di tempo congruo alle mie aspettative tra le mie azioni e la risposta dell'esperienza in realtà virtuale
15	I was able to actively explore or observe the environment during the virtual reality experience	Sono riuscito ad esplorare o osservare attivamente l'ambiente durante l'esperienza in realtà virtuale
16	While I was experiencing virtual reality, I thought about how much I was enjoying it	Mentre svolgevo l'esperienza in realtà virtuale pensavo a quanto mi stava piacendo
17	17. The virtual reality experience did not hold my attention at all (R)	L'esperienza in realtà virtuale non ha per niente attirato la mia attenzione (R)
18	After trying the virtual reality experience for a while, I feel quite competent	Dopo essermi cimentato nell'esperienza in realtà virtuale per un po', mi sento abbastanza competente
19	I am not very good at this virtual reality experience (R)	Questa esperienza in realtà virtuale non mi riesce bene (R)
20	It was important for me to perform the task well during the virtual reality experience.	Era importante per me svolgere bene il compito durante l'esperienza in realtà virtuale
21	I did not put much energy into performing the task during the virtual reality experience (R)	Non ho dedicato molta energia allo svolgimento del compito durante l'esperienza in realtà virtuale (R)
22	I felt very tense while doing this virtual reality experience (R)	Mi sentivo molto teso/a mentre facevo questa esperienza in realtà virtuale (R)
23	I felt comfortable while doing this virtual reality experience	Ero a mio agio mentre facevo questa esperienza in realtà virtuale
24	I think this virtual reality experience is useful for evacuation procedures in case of a fire (electrical)	Penso che questa esperienza in realtà virtuale sia utile per le procedure di evacuazione in caso di incendio (elettrico)
25	I think the virtual reality experience is an important activity	Penso che l'esperienza in realtà virtuale sia un'attività importante

1.4. Perceived Stress during the simulation scale (ad hoc)

**Perceived Stress during the simulation scale**

Instruction: Below you will find some statements relating how you felt during the experience that has just ended. Read each statement carefully and rate your level of agreement on a scale of 1 to 5, where 1 = not at all and 5= very much.		
	English Version	Italian Version
1	I felt unable to control the situation during the simulation	Mi sono sentito/a incapace di controllare la situazione durante la simulazione
2	I felt nervous and stressed during the simulation	Mi sono sentito/a nervoso/a e stressato/a durante la simulazione
3	I felt unable to cope with the procedures during the simulation	Mi sono sentito/a incapace di affrontare le procedure durante la simulazione
4	I felt unable to control situations that irritated me	Mi sono sentito/a incapace di controllare eventuali situazioni che mi hanno irritato/a
5	I felt angry or frustrated about events during the simulation that were beyond my control	Mi sono sentito/a arrabbiato/a o frustrato/a per eventi avvenuti durante la simulazione che erano al di fuori del mio controllo
6	I felt that there were too many things to do during the simulation and that I would not be able to do them all	Ho avuto la sensazione che le cose da fare durante la simulazione fossero troppe e che non sarei riuscito/a a farle tutte

2 Supplementary Results

2.1. Distribution used in the Bayesian models

The errors were modeled with a Poisson distribution (log link), see Figure S1.

Total time with a Gamma distribution (log link), see Figure S2.

The normalized total scores with a Beta regression (logit link), see Figure S3.

The effect found in the Bayesian model, see Figure S4.

Figure S1. Errors distribution

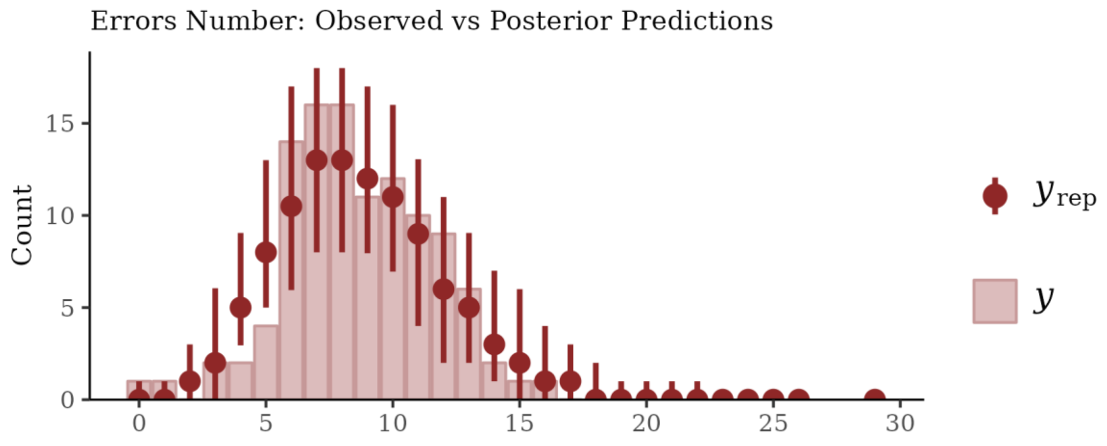

Figure S2. Time distribution

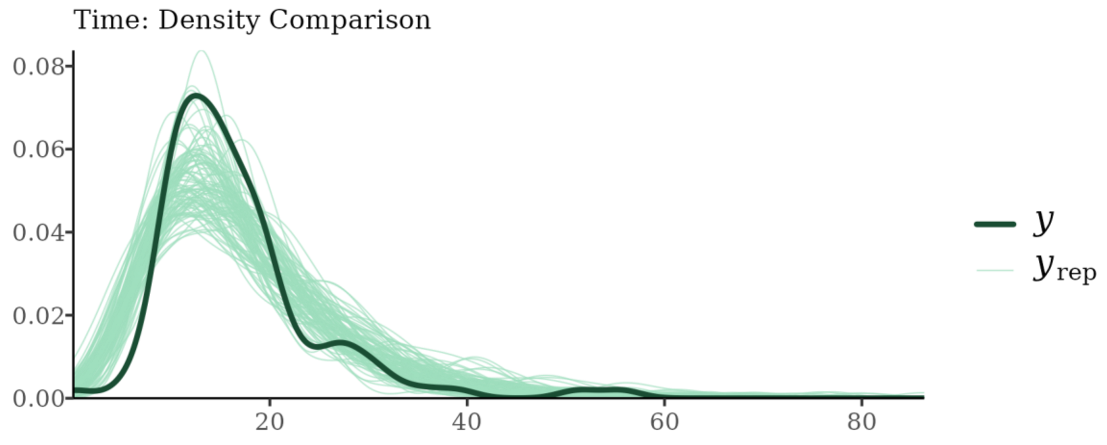

Figure S3. Total score distribution

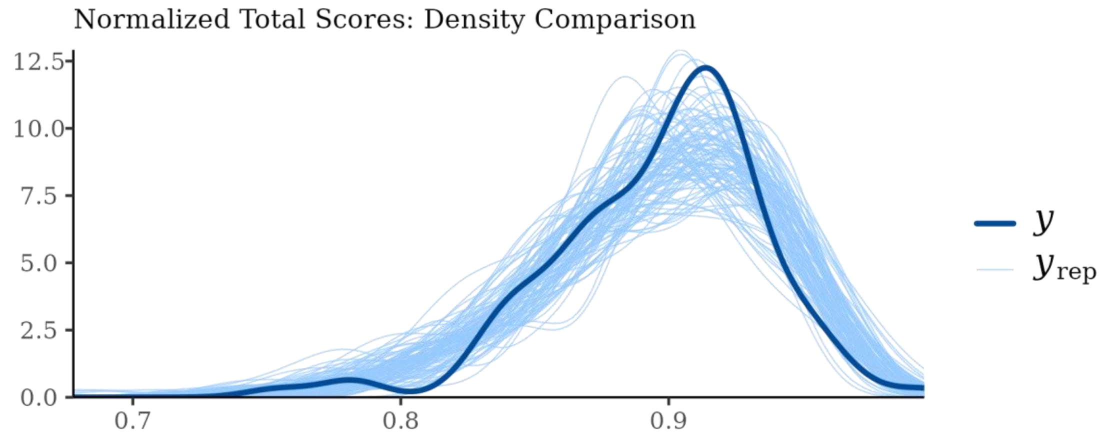

2.2. Study 1b. Correlation matrices

Table S1. Correlations between variables in workers and students.

	1	2	3	4	5	6	7	8	9	10	11	12	13
1. Gender (male)	-												
2. Gender (female)	-	-											
3. Age	.21	-.21	-										
4. Mental rotation test	**.32***	**-.32***	-.22	-									
5. Device familiarity	**.66*****	**-.66*****	-.10	**.47*****	-								
6. Factual knowledge (pre)	.16	-.16	.10	.06	.11	-							
7. Factual knowledge (post)	-.24	.24	-.08	.03	-.24	-.19	-						
8. Evaluation of VR experience	.21	-.21	.25	.12	.28	-.01	-.11	-					
9. Perceived stress	**-.35***	**.35***	-.16	-.25	**-.44****	.01	-.06	**-.64*****	-				
10. Type user (workers)	.17	-.17	**.83*****	-.19	-.10	.17	-.03	.26	-.24	-			
11. Type user (students)	-.17	.17	**-.83*****	.19	.10	-.17	.03	-.26	.24	-1.00***	-		
12. Assessment phase: errors	**-.33***	**.33***	-.21	-.19	**-.30***	.01	.20	**-.42****	**.40****	-.11	.11	-	
13. Assessment phase: time	**-.28***	**.28***	-.13	-.20	**-.34***	-.26	.00	-.13	.20	-.22	.22	**.55*****	-
14. Assssment phase: total score	**.39****	**-.39****	.28	.24	**.36***	.11	-.21	**.37****	**-.32***	.19	-.19	**-.86*****	**-.71*****

Note. Spearman ρ was reported for associations between continuous variables, Phi (φ) was used for dichotomous categorical variables, Cramér's V was used for non-dichotomous categorical variables, and the Kruskal–Wallis test with Cliff's delta was used for associations between continuous and categorical variables. Significant correlations in bold type

Table S2. Correlations between variables in workers

	1	2	3	4	5	6	7	8	9	10	11
1. Gender (male)	-										
2. Gender (female)	-	-									
3. Age	.27	-.27	-								
4. Mental rotation test	.46	-.46	**-.58***	-							
5. Device familiarity	**.74*****	**-.74*****	**-.61***	**.68****	-						
6. Factual knowledge (pre)	.28	-.28	-0.24	**.59***	.35	-					
7. Factual knowledge (post)	-.52	.52	.14	-.11	-.44	-.04	-				
8. Evaluation of VR experience	.14	-.14	-.14	.33	.23	.03	-.09	-			
9. Perceived stress	-.17	.17	.34	-.46	-.30	-.17	-.05	**-.72*****	-		
10. Assessment phase: errors	-.21	.21	.18	-.25	-.23	-.37	.10	-.16	.12	-	
11. Assessment phase: time	-.17	.17	.25	-.25	-.35	-.55	.36	.10	-.22	.38	-
12. Assessment phase: total score	-.02	.02	-.01	.02	-.03	.09	-.01	-.03	.08	.24	-.11

Note. Spearman ρ was reported for associations between continuous variables, Phi (φ) was used for dichotomous categorical variables, Cramér's V was used for non-dichotomous categorical variables, and the Kruskal–Wallis test with Cliff's delta was used for associations between continuous and categorical variables. Significant correlations in bold type

frontiers
